# A Personalized, Interactive, Cognitive Behavioral Therapy–Based Digital Therapeutic (MODIA) for Adjunctive Treatment of Opioid Use Disorder: Development Study

**DOI:** 10.2196/31173

**Published:** 2021-10-08

**Authors:** Björn Meyer, Geri-Lynn Utter, Catherine Hillman

**Affiliations:** 1 GAIA AG Hamburg Germany; 2 Orexo US, Inc Morristown, NJ United States; 3 Central Behavioral Health Norristown, PA United States

**Keywords:** MODIA, opioid use disorder, digital therapeutic, cognitive behavioral therapy, medication-assisted treatment, Broca

## Abstract

**Background:**

Opioid use disorder (OUD) is characterized by the inability to control opioid use despite attempts to stop use and negative consequences to oneself and others. The burden of opioid misuse and OUD is a national crisis in the United States with substantial public health, social, and economic implications. Although medication-assisted treatment (MAT) has demonstrated efficacy in the management of OUD, access to effective counseling and psychosocial support is a limiting factor and a significant problem for many patients and physicians. Digital therapeutics are an innovative class of interventions that help prevent, manage, or treat diseases by delivering therapy using software programs. These applications can circumvent barriers to uptake, improve treatment adherence, and enable broad delivery of evidence-based management strategies to meet service gaps. However, few digital therapeutics specifically targeting OUD are available, and additional options are needed.

**Objective:**

To this end, we describe the development of the novel digital therapeutic MODIA.

**Methods:**

MODIA was developed by an international, multidisciplinary team that aims to provide effective, accessible, and sustainable management for patients with OUD. Although MODIA is aligned with principles of cognitive behavioral therapy, it was not designed to present any 1 specific treatment and uses a broad range of evidence-based behavior change techniques drawn from cognitive behavioral therapy, mindfulness, acceptance and commitment therapy, and motivational interviewing.

**Results:**

MODIA uses proprietary software that dynamically tailors content to the users’ responses. The MODIA program comprises 24 modules or “chats” that patients are instructed to work through independently. Patient responses dictate subsequent content, creating a “simulated dialogue” experience between the patient and program. MODIA also includes brief motivational text messages that are sent regularly to prompt patients to use the program and help them transfer therapeutic techniques into their daily routines. Thus, MODIA offers individuals with OUD a custom-tailored, interactive digital psychotherapy intervention that maximizes the personal relevance and emotional impact of the interaction.

**Conclusions:**

As part of a clinician-supervised MAT program, MODIA will allow more patients to begin psychotherapy concurrently with opioid maintenance treatment. We expect access to MODIA will improve the OUD management experience and provide sustainable positive outcomes for patients.

## Introduction

Opioid use disorder (OUD) is characterized by loss of control of opioid use; recurrent opioid use despite efforts to cut down and despite having persistent physical, psychological, social, or interpersonal problems associated with opioid use; impaired social functioning; craving; tolerance; and withdrawal [1]. Despite attempts in recent years to combat the situation in the United States, the burden of opioid misuse and OUD is a national crisis with substantial public health, social, and economic implications [2]. A 2019 report from the Substance Abuse and Mental Health Services Administration found that in the past year, 9.5 million American adults misused opioids [3]. The same report found that 1.5 million American adults had OUD in the past year [3]. The opioid crisis has led to significant loss of life, with 63%-82% of drug overdose deaths involving 1 or more opioids [4-6]. Drug overdose deaths involving prescription opioids have risen steadily over the past 2 decades, reaching 17,029 in 2017 [7]. Meanwhile, deaths from nonprescription synthetic opioids such as fentanyl have increased exponentially in recent years, from fewer than 5000 in 2013 to 28,466 in 2017 [7]. The opioid crisis also comes with debilitating financial costs. In 2015, the overall economic burden of the opioid crisis was estimated to be US $504 billion [4].

Medication-assisted treatment (MAT) is the current standard treatment for opioid addiction and involves the use of medications, in combination with counseling and behavioral therapies, to provide a “whole-patient” approach to the treatment of OUD [8]. MAT has been demonstrated to reduce illicit opioid use and opioid craving, improve treatment retention, and help sustain recovery [9-11]. One modality of therapy used in the MAT population is cognitive behavioral therapy (CBT), an evidenced-based type of psychotherapy built on the idea that cognitions (eg, thoughts, beliefs, and schemas) and behaviors play a central role in the etiology and maintenance of psychopathology [12]. CBT is considered an evidenced-based approach for the treatment of many psychiatric conditions [12] and has demonstrated added benefit when combined with OUD pharmacotherapies [13-17]. In addition, CBT alone has demonstrated preliminary efficacy in relation to other forms of drug counseling and psychosocial support in patients with OUD [18,19]. Multiple OUD medications—namely methadone, extended-release naltrexone, buprenorphine monotherapy, and buprenorphine/naloxone combination product—are available as part of an MAT program. These drugs are indicated for use as part of a comprehensive treatment plan that includes counseling conducted by a mental health professional and psychosocial support [20-25].

Despite these indications and the demonstrated efficacy of MAT, access to effective counseling, psychotherapy, and psychosocial support is a limiting factor in the treatment of OUD and a significant problem for many patients and physicians. There are an insufficient number of addiction psychiatrists and counselors in the United States, and many clinicians lack the proper training to provide adequate, evidence-based counseling or psychotherapy such as CBT for patients with OUD [26,27]. In a survey of physicians actively prescribing buprenorphine, 93% thought most patients would benefit from counseling, but only 36% reported an adequate number of counselors in their area [28]. Medical providers also lack the financial incentives and training to deliver and coordinate psychological interventions. Current reimbursement models are disproportionately focused on the pharmacotherapy aspect of OUD treatment, with the behavioral component significantly underfunded [29]. Moreover, many reimbursement models do not support care coordination and psychosocial services, and development of models to support MAT delivery are needed [30].

The shortage of counselors likely translates to deficits in psychological intervention because in a survey of 400 patients who were taking buprenorphine, 41% reported not receiving counseling in their first 30 days of treatment [31]. The limited access and use of psychological interventions are likely to continue in the future. Under one scenario analyzed by the National Center for Health Workforce Analysis, multiple provider types, including psychiatrists and substance abuse and behavioral disorder counselors, are predicting a shortage of more than 10,000 full-time equivalent positions by 2025 [32].

In addition to the limited availability of effective counseling services, attitudinal barriers such as stigma can also prevent individuals from seeking or receiving counseling or psychotherapy [33-35]. Patients often worry about how their doctor will react to a disclosure of substance use and potential consequences of having this information in their medical records [34]. These concerns appear somewhat warranted because negative attitudes toward patients with OUD among providers limit access to treatment, harm reduction services, and may lead to the receipt of suboptimal care [33]. Logistical issues, such as busy lifestyles and difficulty traveling, can also complicate access to counseling and prescriptions [36]. Furthermore, barriers to MAT are exacerbated for vulnerable populations, including older people, racial minorities, people who live in rural communities, and those who are homeless, unemployed, or require payment assistance for treatment [33,36-38].

The opioid crisis has placed an enormous burden on the US health care system and has prompted significant support for new and innovative treatment alternatives. One such alternative is digital therapeutics (also discussed under labels such as internet-based interventions, web-based self-help, web-based psychological intervention, and computerized or electronic CBT, among others), an innovative new category of medical mobile apps that help prevent, manage, or treat diseases by delivering therapy through the use of software programs [39]. Digital therapeutics can circumvent barriers to uptake, improve treatment adherence, and enable broad delivery of evidence-based management strategies to meet service gaps [40,41]. Digital therapeutics have been shown to be effective across a broad range of psychiatric conditions, including depression, anxiety, and addictive disorders [42-45]. However, few digital therapeutics have thus far specifically targeted OUD.

A notable digital therapeutics platform for OUD that has been described in the literature is reSET-O, a prescription CBT digital therapeutic intended to be used as an adjunct to outpatient buprenorphine treatment that encompasses contingency management (CM). In an unblinded, controlled clinical trial, addition of reSET-O significantly increased retention in a 12-week treatment program. Although patients were generally compliant with the program, addition of reSET-O did not decrease illicit drug use in comparison with buprenorphine plus CM alone [44]. reSET-O was cleared by the US Food and Drug Administration (FDA) in 2018 [46] for use by patients who are currently under the supervision of a clinician as an adjunct to outpatient treatment that includes transmucosal buprenorphine and CM, validating the potential of digital therapeutics for OUD [47].

Because there is only 1 FDA-cleared digital therapeutic for OUD currently on the market, additional options are needed, especially those that maximize the personal relevance and emotional impact of the interaction to potentially increase learning effects and enhance overall treatment effectiveness. Multiple studies suggest that individually tailored digital interventions tend to be more effective than their nontailored counterparts, possibly because tailoring increases perceived personal relevance, which then leads to more elaborated cognitive processing and greater therapeutic impact [48-50]. MODIA is a novel digital therapeutic that aims to engage patients with OUD in a series of “simulated dialogues” in which a broad range of CBT skills and exercises are conveyed and practiced. The program is designed to tailor the content and style of these CBT skills, as described below, to maximize the relevance to individual patients’ needs and preferences. Here, we describe the development of MODIA with the aim of providing effective, accessible, and sustainable management for patients with OUD.

## Methods

MODIA is a digital therapeutic for the treatment of OUD, which is rooted in evidence-based treatment techniques that are consistent with a CBT framework. It is intended to be used as part of a clinician-supervised MAT program. MODIA tailors content to the individual user, providing a personalized and interactive psychotherapy intervention that engages end users in CBT exercises and aims to empower them with skills to cope with cravings, withdrawal symptoms, potential trigger situations, and emotional symptoms accompanying OUD (eg, anxiety and depression). MODIA also allows users to develop a customized relapse prevention plan that encompasses risk behaviors, triggers, cravings, and coping strategies on the basis of patient inputs collected throughout the module exercises.

**Figure 1:**
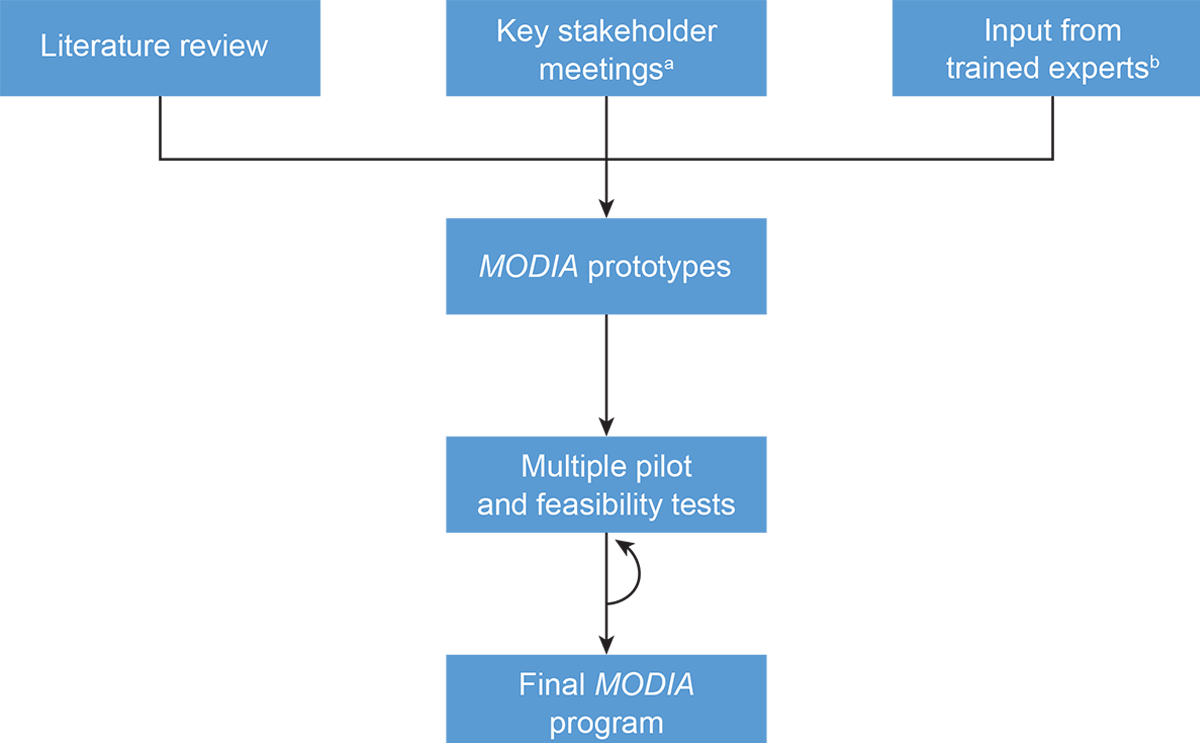
MODIA Development Process (September 2019 to January 2021). a: Key stakeholders included experienced physicians, OUD treatment specialists, and patients at various stages of recovery. b: Trained experts included clinical psychologists, CBT therapists, software engineers, experienced physicians, and OUD treatment specialists. OUD, opioid use disorder; CBT cognitive behavioral therapy.

MODIA was developed by a multidisciplinary, international development team associated with GAIA AG in Hamburg, Germany. The development process followed a framework developed by GAIA over the course of more than a decade and is generally consistent with models such as the patient-focused, person-centered approach described by Yardley et al [51-54]. The MODIA development team included several licensed clinical psychologists and CBT therapists, software engineers, creative writers, graphic artists, and professional speakers. Prior to the development of the program, relevant treatment manuals, intervention descriptions, guidelines, patient reports, and trial results were reviewed by the development team (Figure 1). Several members of the development team (including BM and GU) also met in person on several occasions with experienced physicians, OUD treatment specialists, and patients at various stages of recovery. Some of these meetings took place in areas that are most severely affected by the current opioid crisis, including the Kensington neighborhood in Philadelphia, Pennsylvania. In the spirit of participant observation [55], members of the development team also attended a Narcotics Anonymous meeting in this neighborhood and had informal conversations with a variety of patients and MAT providers. Throughout the development process, several small pilot and feasibility evaluations were conducted with prototypes of the program, and results were used to refine and improve the program. Because these evaluations were part of the commercial product development process rather than academic studies, their specific results are not reported here; however, brief summaries are available upon request from the authors. On the basis of the findings of the development team, a broad range of behavior change techniques were incorporated into MODIA and are described in Table 1 [56].

**Table 1:** Behavior change techniques included in MODIA.

Technique	Representative examples
Action planning and mental contrasting	Envisioning how to act in high-risk situations Envisioning how to overcome obstacles
Avoidance	Reducing exposure to cues Reflecting on people, places, and things associated with prior opioid use Restructuring the social environment to support recovery
Behavioral substitution	Encouraging engagement in alternative behaviors in high-risk situations
Credible source	Explaining how specific claims and techniques have been validated in well-designed studies
Decisional balance exercises	Reflection of advantages and disadvantages of using opioids
Direct therapeutic advice	How to use simple therapeutic techniques
Functional analysis	Identifying individual antecedents and consequences of opioid use How to change sequences of triggers
Goal setting and progress review	Expression of commitment to abstinence Normalization and validation of relapse
Homework	To practice a variety of cognitive behavioral therapy techniques covered in each chat
Humor	“Bruce the parrot” verbalizing unhelpful thoughts to convey “cognitive defusion”
Mental imagery	Envisioning a “healthy future self” Guided meditation
Metaphors and images	Cravings as “ocean waves” Unhelpful thoughts as “leaves floating on a stream”
Problem solving	Teaching effective skills and general problem-solving strategies
Psychoeducation	Cognitive behavioral therapy techniques Basic neurobiological processes underlying opioid dependence Role of exercise, nutrition, and sleep hygiene in recovery
Reward	Praise for continued program engagement “Stars and crowns” (images) to reward progress
Simulated role-plays	Resisting social pressures to use drugs Assertive communication
Self-monitoring and feedback	Interactive self-report questionnaires
Self-talk	Teaching patients to practice internal monologue to support recovery
Storytelling	Presentation of fictional cases
Therapeutic writing	Writing about personally relevant issues
Validation	Patients are not judged for their behavior Patients’ efforts are recognized and valued

## Results

MODIA uses proprietary software technology (Broca) that dynamically tailors content to the users’ responses. This software is the basis for several other digital therapeutic programs developed by this group and has been shown to be effective in multiple clinical trials [42,52-54,57-59]. Broca-based programs utilize an interactive approach in which the patient selects at least 1 option from predetermined menus within the program. Patients’ responses dictate what content is subsequently presented, creating a “simulated dialogue” experience between the patient and program. On the basis of patients’ responses, various aspects of the intervention are customized to match individual needs and preferences; for example, content is conveyed in either a more empathic/warmer style or a more directive/irreverent style; patients can choose to skip certain sections or case examples; and they are offered brief exercises relevant to their situation (eg, a brief exercise on coping with shame is offered only to patients who indicate that they have felt a sense of shame and would like to learn how to cope with it). MODIA uses simple, colloquial language to enhance user engagement. The purpose of presenting therapeutic content in an informal, dialogical fashion is to simulate key characteristics of human therapeutic interactions, such as responsiveness to patient requirements, personal relevance, empathy, and the therapeutic alliance. Consistent with this approach, evidence has shown that the quality of the therapeutic alliance with a Broca-based digital therapeutic predicts therapeutic improvement [60] and that individually tailored digital interventions tend to outperform their nontailored counterparts [49].

**Figure 2:**
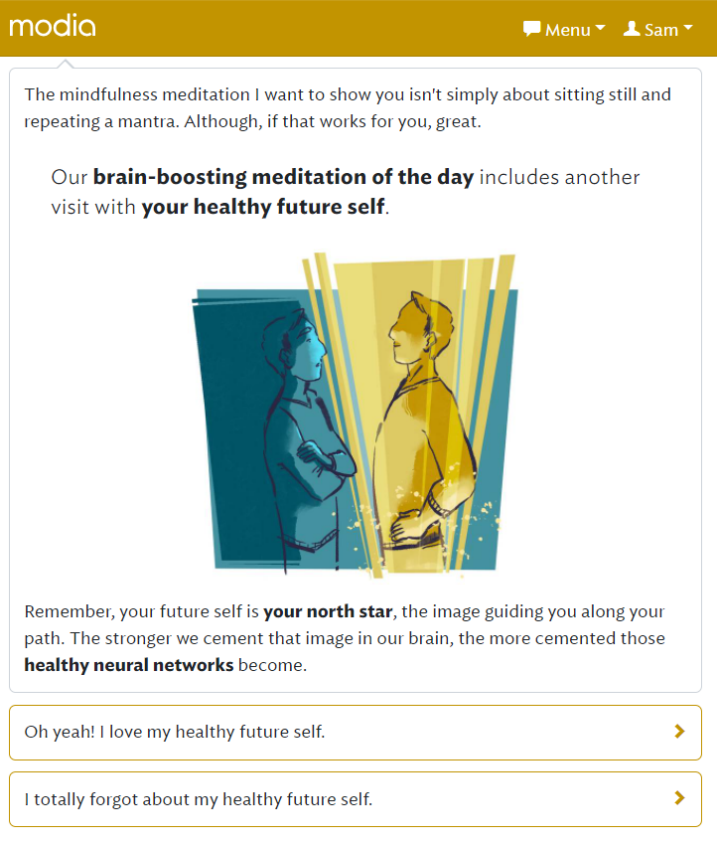
MODIA screenshot 1.

**Figure 3:**
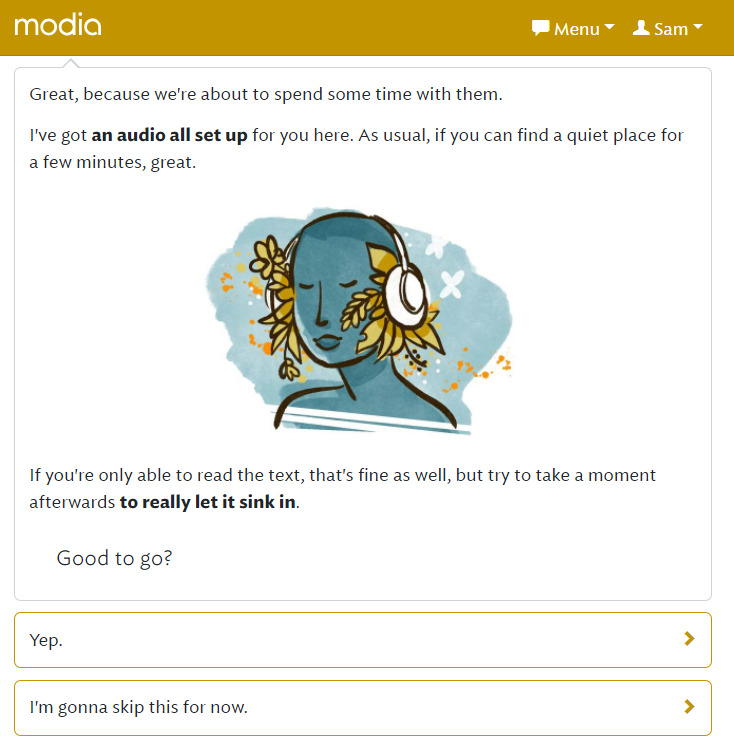
MODIA screenshot 2.

**Figure 4:**
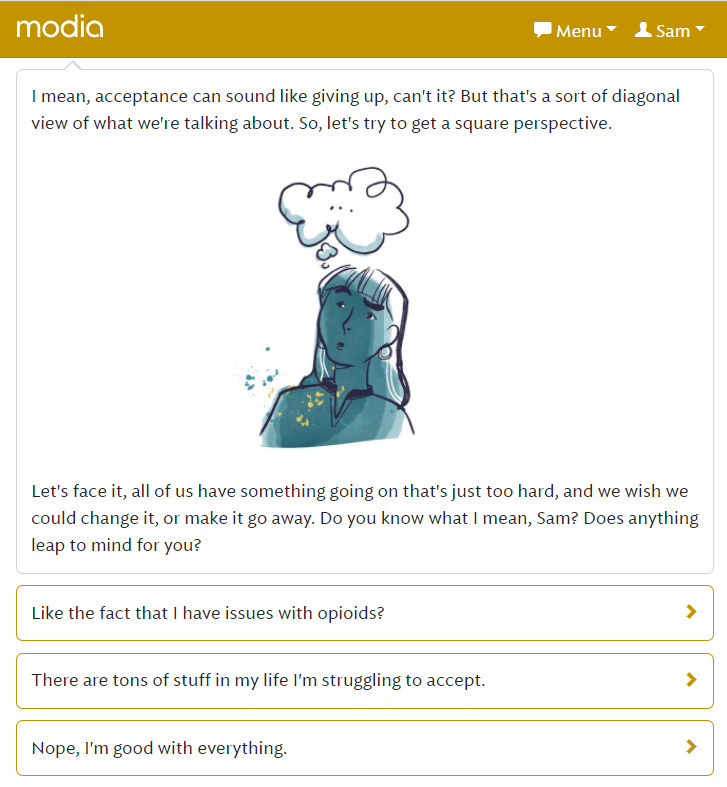
MODIA screenshot 3.

**Figure 5:**
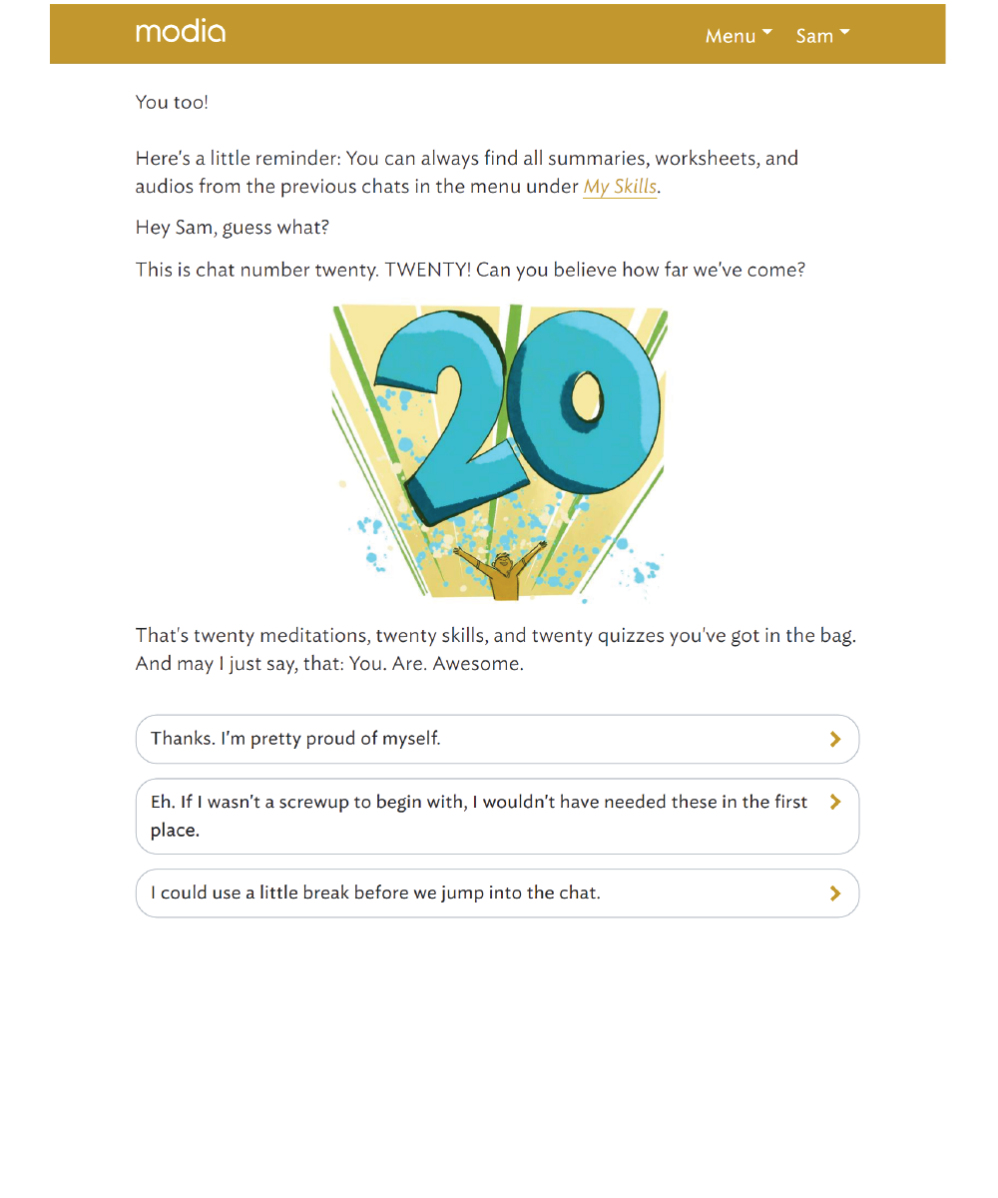
MODIA screenshot 4.

**Figure 6:**
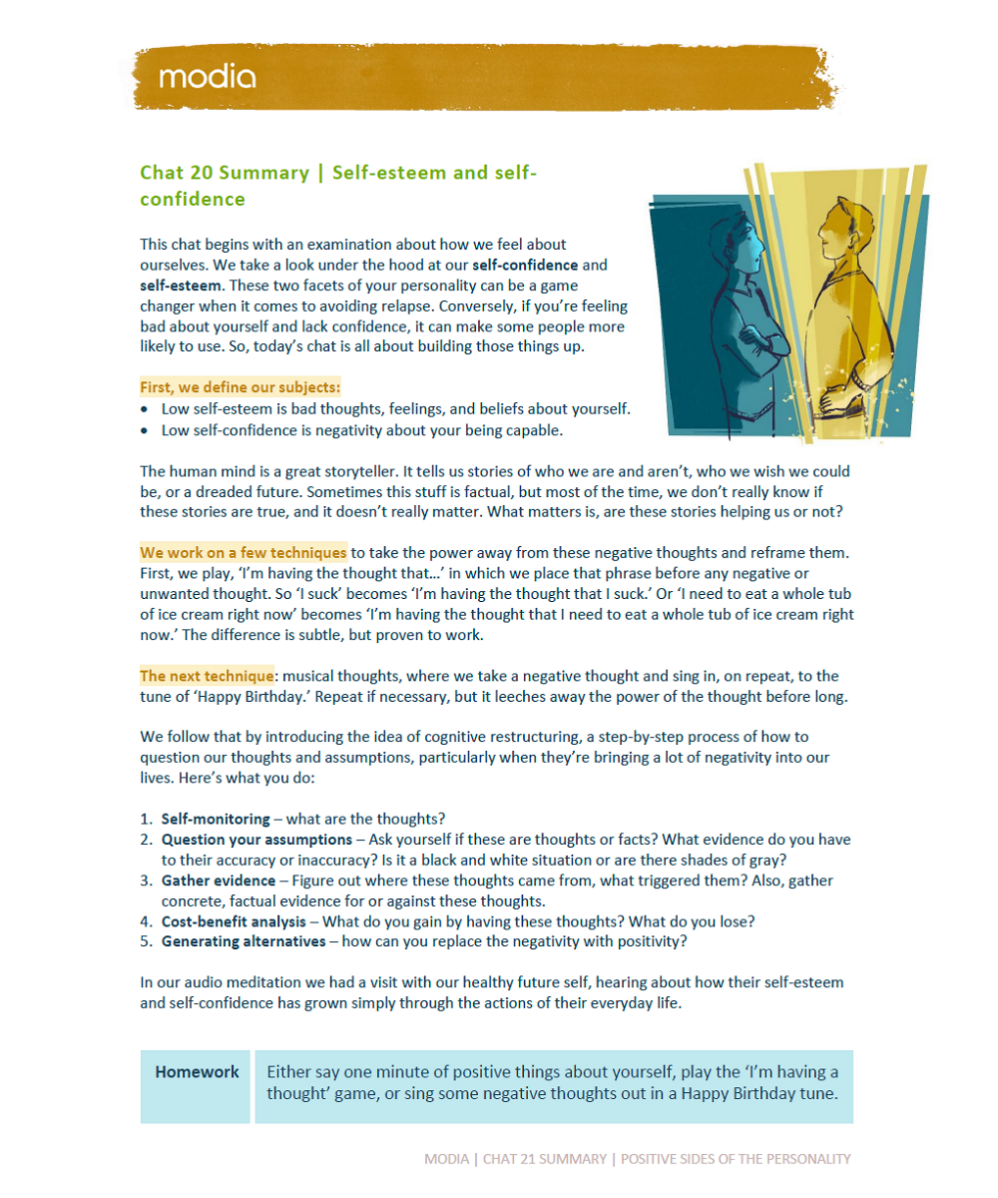
MODIA screenshot 5.

Before using MODIA, patients receive a 12-digit personal registration code. After entering this code and accepting the program’s terms and conditions, patients are asked to enter their email and set a password, which they can use to access the program for 180 days on any suitable device, including smartphones and desktop, laptop, or tablet computers. The MODIA program comprises 24 modules or “chats.” The term “chat” is used to be consistent with the idea that the program engages in a simulated therapeutic dialogue with the patient, which is a central metaphor guiding the patient’s experience. Patients are instructed to work independently by completing 1 to 2 chats per week. Each chat can be completed in approximately 15 to 30 minutes, depending on factors such as reading speed, selection of optional audio recordings, and individual response options or paths through the program. In addition to these chats, MODIA also includes brief motivational text messages that are sent regularly to prompt patients to use the program and help them transfer therapeutic techniques into their daily routines. Screenshots that convey the look and feel of MODIA are shown in Figures 2-6.

The chats are grouped into 4 clusters. Table 2 shows the chat topics, goals, and content outlines for clusters 1 and 2 as examples of the content found within a cluster; content outlines for all 4 clusters are presented in Multimedia Appendix 1. In brief, the first cluster is “Basic Techniques and Principles,” in which patients are oriented to the program, learn about the neurobiology of opioid dependence, and acquire basic CBT skills. In the “Learning Psychological Flexibility Skills” cluster, patients are taught 6 core skills to increase “psychological flexibility” or the capacity to tolerate distress [61,62]. In the third cluster, “Applying Therapeutic Skills to Important Life Domains,” patients learn to apply the techniques they have learned to various relevant life domains such as interpersonal relationships, coping with depression or anxiety/worries, anger management, and insomnia. Finally, the “Facilitating Personal Growth and Development: Solidifying Your Healthy Self-Identity” cluster emphasizes the strengths, talents, and personal resources of the patient. Patients are taught to practice compassion, engage in exercises that build self-esteem and confidence, discover personal strengths, and cope successfully with slips and relapses. Building life skills such as these can help patients manage stressful situations and environmental cues that may trigger cravings and relapse. Furthermore, skills that patients develop through CBT are likely to remain even after treatment has ceased [63]. Patient-friendly language (ie, lay terms rather than medical jargon) is used in the program to describe the clusters and chats.

**Table 2:** Content outline of MODIA clusters 1 and 2.

Topic (chat title)^a^			Main goal of the chat		Content outline^b^
**Cluster 1: “Basic techniques and principles”**					
	1. Introduction to MODIA (“Meet and greet”)	Orient and engage patients; provide basic education and motivation to continue using MODIA.		Introduction to the program’s function and purpose Facilitating hope and positive expectancies Interactive exploration of patient’s background Risk and safety information Recommendations for optimal program use	
	2. Enhancing motivation (“Taking the measurements”)	Build motivation by encouraging patients to reflect on the advantages of abstaining and the disadvantages of continuing to use opioids.		Interactive exploration of current motivation to stop using opioids Exploration of motivational stages of change Enhancing motivation by building awareness of personal reasons for and ability to change	
	3. Functional analysis (“The bird’s eye view”)	Empower patients to gain greater clarity on trigger situations and teach simple techniques to improve their ability to resist urges to use opioids.		Build awareness of personal high-risk situations and triggers Introduction to functional analysis Simple techniques to cope with triggers Audio mental imagery/mindfulness meditation exercise to build skills to resist triggers and cravings	
	4. Behavioral coping with triggers (“Look over there!”)	Empower patients by teaching them how to identify and avoid high-risk situations and use simple behavioral techniques to cope with such situations.		Interactive functional analysis and structured assessment of: Personal trigger situations Automatically elicited thoughts and feelings Typical behaviors in high-risk situations Short-term consequences Negative long-term consequences Interactive exploration of potential approaches to altering contingencies; using behaviors for distraction coping	
	5. Cognitive coping with triggers (“The stranger in the mirror”)	Empower patients by teaching them simple methods targeting cognitions that increase risk for opioid use.		Use of mental strategies rather than physical distraction activities to cope with triggers or urges to use Audio exercise: revisiting the “healthy future self” Fictional case example to illustrate successful and unsuccessful coping Interactive exploration of cognitive coping techniques Recognizing common cognitive distortions	
	6. Review of first cluster (“Let’s get physical”)	Review previously learned CBT^c^ techniques and educate patients on role of healthy lifestyle in recovery.		Review of key techniques from previous five “chats” Integrating the CBT techniques to encourage having a healthy lifestyle Interactive exploration of the role of nutrition in opioid dependence Interactive exploration of sleep habits and review of principle of sleep hygiene Audio exercise: mental imagery to review key techniques from Cluster 1	
**Cluster 2: “Learning psychological flexibility skills”**					
	7. Defusion and emotional distancing (“The defusion solution”)	Teach patients to learn “defusion” techniques to distance themselves from unhelpful thoughts and feelings.		Introduction to the core topic of Cluster 2: “psychological flexibility” Overview and interactive exploration of the 6 components of PF^d^ (eg, defusion, acceptance, presence, self-discovery, values, and committed action)	
	8. Acceptance and distress tolerance (“The acceptance conundrum”)	Teach patients acceptance skills to improve distress tolerance while remaining committed to recovery-related goals.		Introduction to acceptance as a key psychological flexibility technique Interactive exploration of aversive thoughts and feelings Experiential exercise to illustrate difficulties with thought suppression Therapeutic metaphors to convey the principle of acceptance Introduction of the acceptance and commitment therapy concept and the skill of “willingness” Mental imagery story-based exercise to experience and practice willingness	
	9. Mindfulness and presence (“Enter the Buddha”)	Teach patients mindfulness techniques to reduce stress and improve coping with cravings, urges to use, and other aversive mental and emotional experiences.		Brief step-by-step guided experiential mindfulness exercise Mindfulness meditation exercises Guided mindfulness exercise Fictional case examples to convey the personal relevance of mindfulness meditation	
	10. Self-discovery (“Who am I?”)	Teach patients self-discovery skills to help them cope with high-risk situations and improve their general ability to remain committed towards healthy life goals.		Introduction to the 3 facets of self-discovery: the “conceptual self,” contacting the “stream of consciousness,” and “the observing self” Invitation to engage in expressive writing exercise Fictional case example to illustrate expressive writing; exercises to discover and observe the stream of consciousness Experiential exercise on the “observing self”	
	11. Values clarification (“The best values”)	Teach patients to clarify valued life directions to orient them toward a healthy life “beyond opioid dependence” and thereby support their recovery goals.		Mental imagery exercise (“revisiting your healthy future self”) Interactive introduction to personal values clarification as a key component of psychological flexibility Fictional case example to illustrate the relevance of personal values Interactive review of importance and time investment with regard to common core values Interactive exploration of relevance of personal values in the context of opioid dependence	
	12. Commitment to healthy actions (“Do it!”)	Teach patients “behavioral commitment” techniques to support their efforts to achieve healthy recovery goals.		Introduction to the “committed action” psychological flexibility facet Review of potential obstacles that might prevent patient from pursuing core values Fictional story to illustrate the concept of “SMART” (specific, measurable, adaptive, realistic, and time-framed) goals Exploration of simple strategies to increase commitment to value-consistent actions Mindfulness-based audio recording on committed action	

^a^Please see Multimedia Appendix 1 for a full outline of all 4 MODIA content clusters.

^b^Most “chats” also include a brief review of the patients’ emotional state, a review quiz, and homework assignment.

^c^CBT: cognitive behavioral therapy.

^d^PF: psychological flexibility.

Although MODIA is aligned with CBT principles, it was not designed to present any 1 specific CBT treatment in digital format; rather, it uses a broad range of evidence-based behavior change techniques drawn from CBT, mindfulness, acceptance and commitment therapy (ACT), and motivational interviewing (MI) (Table 2). Mindfulness and ACT encourage patients to observe and accept negative thoughts and emotions without judgment, and MI encourages patients to articulate their reasons to change [64-66]. Techniques learned from these therapeutic modalities focus on increasing patient psychological flexibility or distress tolerance to support patients’ efforts to achieve recovery from OUD, consistent with recent evidence demonstrating the effectiveness of such techniques for substance use disorders [62].

## Discussion

### Principal Findings

Innovative, effective, and evidence-based management strategies are needed to address the opioid crisis, the substantial burden of OUD, and the limitations in access to effective counseling and care for individuals with OUD. To this end, a multidisciplinary team developed MODIA on the basis of a review of the relevant literature and in-person meetings with key stakeholders to offer individuals with OUD a tailored, interactive digital psychotherapy intervention. The purpose of this custom-tailored individualization and personalization is to maximize the personal relevance and emotional impact of the interaction because these aspects may increase learning effects and enhance overall treatment effectiveness [49,67]. The content of MODIA is CBT-consistent but also unique and innovative, utilizing psychological flexibility-based techniques that may be particularly effective in the treatment of substance use disorders [62]. Moreover, MODIA integrates principles and techniques from MI, which encourage the patient to build awareness of personal reasons to change, to effectively direct them toward change [66], and to acquire skills for enhanced psychological flexibility, which can be regarded as a cornerstone of mental health [61]. Notably, MODIA does not use a financially based contingency management element because this may hinder product adoption at both a health care professional (HCP) level and an insurer level. In addition, contingency management may create perverse patient incentives if rewards are designed to reinforce program use rather than recovery.

MODIA is also unique in that it adapts content on the basis of user input, enabling the delivery of an individualized therapeutic experience. MODIA is intended to alleviate barriers to psychological interventions and enable ready access to effective counseling for those who may not have the opportunity to retain counseling services. The self-directed nature of MODIA allows patients to complete the program on their own time and at their own pace without additional oversight by a therapist or counselor. This aspect of MODIA is aided by self-rated questionnaires that are embedded throughout the program and allow for self-monitoring of symptoms and progress. Although MODIA is intended to be used under guidance from a MAT prescriber, MODIA respects patient privacy and is not designed to report symptoms to the patients’ HCPs.

### Limitations

Although MODIA was developed with the intention of lowering barriers to psychological interventions, it is not without limitations. Like other digital therapeutics, MODIA requires an internet connection and a suitable device. Hence, those with limited access to the necessary technology may not be able to use the program. In addition, while multiple randomized controlled trials have demonstrated the clinical value of Broca-based programs using the simulated dialogue approach, some patients may require more intensive or other forms of psychological support.

MODIA is being brought to market under the FDA COVID guidance for industry. MODIA is intended to provide digital CBT for patients with OUD, 18 years of age or older, as a part of a clinician-supervised MAT program for OUD. MODIA is a prescription-only device to be ordered by a clinician. MODIA has not been clinically tested and may therefore have unknown benefits and risks.

### Conclusions

A multidisciplinary team of experts developed MODIA—a fully automated, custom-tailored digital therapy for the management of OUD. As part of a clinician-supervised MAT program, MODIA will allow more patients to begin psychotherapy at the same time they start opioid maintenance treatment. We expect that access to MODIA will improve the MAT experience and provide sustainable positive outcomes for patients with OUD. A randomized controlled trial will be conducted in the future to evaluate the efficacy of MODIA. Additional future studies may evaluate the long-term effects of MODIA; impact on treatment engagement, adherence, and early termination; as well as intervention effects on secondary outcomes such as mental health–related quality of life.

## Appendix

Multimedia Appendix 1 Content outline of the 24 MODIA "chats."

## Abbreviations

ACT: acceptance and commitment therapy
CBT: cognitive behavioral therapy
CM: contingency management
FDA: Food and Drug Administration
HCP: health care professional
MAT: medication-assisted treatment
MI: motivational interviewing
OUD: opioid use disorder
