# Supplementary material for: A Personalized, Interactive, Cognitive Behavioral Therapy–Based Digital Therapeutic (MODIA) for Adjunctive Treatment of Opioid Use Disorder: Development Study
Source: JMIR Ment Health. 2021 Oct 8;8(10):e31173. doi: 10.2196/31173 (PMC8538017; doi:10.2196/31173)
Supplement: Multimedia Appendix 1 [file mental_v8i10e31173_app1.pdf]

**Supplementary Table. Content outline of the 24 MODIA "chats"**

| Topic (chat title) | Main goal of chat | Content outline[a] |
|---|---|---|
| **Cluster 1: "Basic techniques and principles"** | | |
| 1. Introduction to MODIA ("Meet and greet") | Orient and engage patients; provide basic education and motivation to continue using MODIA. | • Introduction to the program's function and purpose<br>• Facilitating hope and positive expectancies<br>• Interactive exploration of patient's background<br>• Risk and safety information<br>• Recommendations for optimal program use |
| 2. Enhancing motivation ("Taking the measurements") | Build motivation by encouraging patients to reflect on the advantages of abstaining and the disadvantages of continuing to use opioids. | • Interactive exploration of current motivation to stop using opioids<br>• Exploration of motivational stages of change<br>• Enhancing motivation by building awareness of personal reasons for and ability to change |
| 3. Functional analysis ("The bird's eye view") | Empower patients to gain greater clarity on trigger situations and teach simple techniques to improve their ability to resist urges to use opioids. | • Build awareness of personal high-risk situations and triggers<br>• Introduction to functional analysis<br>• Simple techniques to cope with triggers<br>• Audio mental imagery/mindfulness meditation exercise to build skills to resist triggers and cravings |
| 4. Behavioral coping with triggers ("Look over there!") | Empower patients by teaching them how to identify and avoid high-risk situations and use simple behavioral techniques to cope with such situations. | • Interactive functional analysis and structured assessment of:<br>  ○ Personal trigger situations<br>  ○ Automatically elicited thoughts and feelings<br>  ○ Typical behaviors in high-risk situations<br>  ○ Short-term consequences<br>  ○ Negative long-term consequences<br>• Interactive exploration of potential approaches to altering contingencies; using behaviors for distraction coping |
| 5. Cognitive coping with triggers ("The stranger in the mirror") | Empower patients by teaching them simple methods targeting cognitions that increase risk for opioid use. | • Use of mental strategies rather than physical distraction activities to cope with triggers or urges to use<br>• Audio exercise: revisiting the "healthy future self"<br>• Fictional case example to illustrate successful and unsuccessful coping<br>• Interactive exploration of cognitive coping techniques<br>• Recognizing common cognitive distortions |
| 6. Review of first cluster ("Let's get physical") | Review previously learned CBT techniques and educate patients on role of healthy lifestyle in recovery. | • Review of key techniques from previous five "chats"<br>• Integrating the CBT techniques to build a healthy lifestyle<br>• Interactive exploration of the role of nutrition in opioid dependence<br>• Interactive exploration of sleep habits and review of principle of sleep hygiene |

| | | |
|---|---|---|
| | | • Audio exercise: mental imagery to review key techniques form Cluster 1 |
| **Cluster 2: "Learning psychological flexibility skills"** | | |
| 7. Defusion and emotional distancing ("The defusion solution") | Teach patients to learn "defusion" techniques to distance themselves from unhelpful thoughts and feelings. | • Introduction to the core topic of Cluster 2: "psychological flexibility" (PF)<br>• Overview and interactive exploration of the six components of PF (e.g., defusion, acceptance, presence, self-discovery, values, committed action) |
| 8. Acceptance and distress tolerance ("The acceptance conundrum") | Teach patients acceptance skills to improve distress tolerance while remaining committed to recovery-related goals. | • Introduction to acceptance as a key PF technique<br>• Interactive exploration of aversive thoughts and feelings<br>• Experiential exercise to illustrate difficulties with thought suppression<br>• Therapeutic metaphors to convey the principle of acceptance<br>• Introduction of the ACT concept and skill of "willingness"<br>• Mental imagery story-based exercise to experience and practice willingness |
| 9. Mindfulness and presence ("Enter the Buddha") | Teach patients mindfulness techniques to reduce stress and improve coping with cravings, urges to use, and other aversive mental and emotional experiences. | • Brief step-by-step guided experiential mindfulness exercise<br>• Mindfulness meditation exercises<br>• Guided mindfulness exercise<br>• Fictional case examples to convey the personal relevance of mindfulness meditation |
| 10. Self-discovery ("Who am I?") | Teach patients self-discovery skills to help them cope with high-risk situations and improve their general ability to remain committed towards healthy life goals. | • Introduction to the three facets of self-discovery: the "conceptual self," contacting the "stream of consciousness," and "the observing self"<br>• Invitation to engage in expressive writing exercise<br>• Fictional case example to illustrate expressive writing; exercises to discover and observe the stream of consciousness<br>• Experiential exercise on the "observing self" |
| 11. Values clarification ("The best values") | Teach patients to clarify valued life directions to orient them toward a healthy life "beyond opioid dependence" and thereby support their recovery goals. | • Mental imagery exercise ("revisiting your healthy future self")<br>• Interactive introduction to personal values clarification as a key component of psychological flexibility<br>• Fictional case example to illustrate the relevance of personal values<br>• Exploration of values can be identified<br>• Interactive review of importance and time investment with regard to common core values<br>• Interactive exploration of relevance of personal values in the context of opioid dependence |
| 12. Commitment to healthy actions ("Do it!") | Teach patients "behavioral commitment" techniques to support their efforts to achieve healthy recovery goals. | • Introduction to the "committed action" PF facet<br>• Review of potential obstacles that might prevent patient from pursuing core values<br>• Fictional story to illustrate the concept of "SMART" goals (specific, measurable, adaptive, realistic, time-framed) |

| | | |
|---|---|---|
| | | • Exploration of simple strategies to increase commitment to value-consistent actions<br>• Mindfulness-based audio recording on committed action |
| **Cluster 3: "Applying therapeutic skills to important life domains"[b]** | | |
| 13. Interpersonal relationships ("Circles of friends") | Empower patients by improving their ability to cultivate harmonious relationships that support their recovery goals and reduce risk for relapse. | • Interactive exploration of the relevance of interpersonal relationship to opioid dependence<br>• Identification of problematic relationships and perceived reasons for problems<br>• Simulated role-plays to apply acceptance and willingness skills to relationships and to practice resisting social triggers<br>• Interactive exploration of relationships that might contribute to opioid use<br>• Interactive review of assertive communication techniques<br>• Mental imagery exercise on helpful and unhelpful relationships |
| 14. Depression ("The deepest shade of blue") | Empower patients by teaching and rehearsing skills to reduce dysphoria and depression, which can interfere with the recovery process. | • Explanation of how opioid dependence increases depression risk, CBT principles, and how CBT can help reduce depression<br>• Interactive review of current depression symptoms<br>• Recommendation to contact healthcare professional or suicide prevention lifeline if needed<br>• Explanation and discussion of key behavioral activation principles<br>• Activity ideas for different valued life domains<br>• Clarification of commitment and opportunity to declare high or low motivation<br>• Identifying and overcoming typical cognitive-affective obstacles in behavioral activation<br>• Applying acceptance- and defusion-based techniques to cope with unhelpful cognitions<br>• Applying psychological flexibility skills to overcome depression |
| 15. Anxiety and persistent worries ("Chewing your nails") | Empower patients by teaching and rehearsing CBT skills for successful anxiety management, which also supports their recovery goals. | • Interactive exploration on anxiety and relevance to opioid dependence<br>• Exploration of personally relevant worries, fears, and anxieties<br>• Experiential exercise: conjuring up anxious thoughts/feelings and applying psychological flexibility techniques to them<br>• Interactive review of key CBT strategies for coping with anxiety<br>• Applying defusion techniques to anxiety<br>• Alternative mental imagery for defusion of anxious thoughts and feelings<br>• Mental imagery exercise on the "healthy future" self who illustrates applying willingness/acceptance techniques to coping with anxiety |
| 16. Anger management ("Seeing red") | Empower patients by teaching and rehearsing effective therapeutic | • Interactive exploration of the personal relevance of anger, frustration, and irritability |

| | | |
|---|---|---|
| | techniques to manage anger and irritability, which could interfere with recovery progress. | • Fictional case example to illustrate how anger is elicited from a CBT perspective<br>• Identifying factors that facilitate anger<br>• Using psychological flexibility techniques to manage anger<br>• Review of a mnemonic to manage anger<br>• Using forgiveness and empathy in anger management |
| 17. Sleep problems and insomnia<br>("Perchance to dream") | Empower patients by teaching and rehearsing effective CBT skills for overcoming insomnia and improving the quality of sleep. | • Discussion of insomnia in the context of opioid dependence<br>• Brief questionnaire on potential sleep problems<br>• CBT strategies for better sleep<br>• "Sleep meditation" to facilitate falling asleep<br>• Interactive guided discovery of CBT sleep principles<br>• Discussion of other treatment options for insomnia (e.g., medication) |
| 18. Problem-solving skills<br>("Checkmate") | Empower patients by teaching them general and broadly applicable techniques for problem-solving across a range of life domains. | • Introduction to problem-solving as a universal CBT technique<br>• Discussion of systematic sequence in problem-solving: problem definition, setting realistic and attainable goals, identifying potential obstacles and strategies for overcoming them<br>• Discussion and review of final problem-solving steps: rank-ordering plans, putting best plan into action<br>• Audio recording exercise: mindful appreciation of past problem-solving successes and overall progress<br>• Brief review of the "STOP method" and application to problem-solving<br>• Simulated role-play to apply the "STOP method" in a high-risk situation that could lead to opioid use |
| **Cluster 4: "Facilitating personal growth and development: solidifying your healthy self-identity"** | | |
| 19. Compassion and self-compassion<br>("Because I care") | Empower patients by teaching them effective compassion and self-compassion skills to help with successful coping with high-risk situations and recovery setbacks. | • Overview of positive psychology and personal growth<br>• Introduction to compassion<br>• Guided discovery of the personal relevance of compassion<br>• Interactive discussion on rationale for compassion training<br>• Mental imagery experiential exercise on "feeling compassion toward others and yourself"<br>• Guided discovery exercise on compassion |
| 20. Self-esteem and confidence<br>("Hey, good lookin'") | Improve patients' sense of self-esteem and self-confidence to further support their ability to achieve their recovery goals. | • Guided discovery of the personal relevance of self-esteem and self-confidence<br>• Discussion of research on self-esteem/confidence in the context of opioid dependence<br>• CBT definition of self-esteem<br>• Differentiating self-esteem from self-confidence<br>• Guided discovery of low self-esteem/confidence |

| | | |
|---|---|---|
| | | • How psychological flexibility techniques can be applied to low self-esteem/confidence; guided discovery and simulated role-play of other defusion techniques<br>• Guided discovery on the detrimental effect of upward social comparison on self-esteem<br>• Mental imagery exercise (“how the healthy future self does not buy into self-disparaging thoughts”) |
| 21. Discovering personal strengths<br>(“Look at those assets”) | Empower patients by focusing their attention on personal strengths and resources, which could help them structure their lives in a healthy manner “beyond opioid dependence.” | • Introduction to discovering personal strengths rather than dwelling on problems/pathology<br>• Mental imagery exercise (“reflections of your strengths in the waterfall”)<br>• Storytelling mental imagery exercise based on the Values in Action (VIA) classification of strengths and virtues<br>• Guided discovery of potential personality strengths across six domains (wisdom, courage, humanity, justice, temperance, transcendence)<br>• Mental imagery exercise: revisiting “people, places, and things” with greater awareness of personal strength |
| 22. Cultivating the healthy future self<br>(“Your healthy future self”) | Empower patients by strengthening their sense of conviction (or self-efficacy) that they can reach their recovery goals and cope successfully with obstacles they might encounter. | • Praise and reinforcement of progress; guided discovery: “revisiting decisional balance”<br>• Revisiting the mental image of the “healthy future self”<br>• Guided mental imagery exercise: “reflecting on your progress, obstacles and setbacks, and the healthy future self”)<br>• Therapeutic writing exercise<br>• Assessing confidence for reaching healthy future self<br>• Anticipating obstacles and setback<br>• Mental rehearsal of potential coping strategies |
| 23. Anticipating relapses<br>(“Relapse”) | Empower patients by teaching them to identify early signs of relapse and respond effectively. | • Introduction to preventing and responding to relapse<br>• Interactive review of different stages of relapse: emotional, mental, and physical<br>• Guided discovery: detecting and responding constructively to signs of emotional, mental and physical relapse<br>• Fictional case example of a person who has relapsed and coped<br>• Audio recording mental imagery exercise: “Urge-surfing in the presence of your healthy future self” |
| 24. Grand review and wrap-up<br>(“Blast off to the future! Thank you!”) | Empower patients by providing an effective review of key techniques encountered throughout the program; providing a sense of closure that supports patients’ confidence and ability to | • Introducing the “memory palace” technique to rehearse key techniques from previous modules<br>• Guided mental imagery: “a visit to the memory palace with a goodbye-party”<br>• Final praise for having finished the program |

| | | |
|---|---|---|
| | continue successfully on their journey to sustained recovery. | • Empathic farewell |

[a]Most clusters also include a brief review of the patients' emotional state, a review quiz, and homework assignment

[b]The sequence of modules in Cluster 3 is not fixed; patients are encouraged to apply CBT skills to relevant problem areas; automated suggestion to start with one particular domain (patients can opt to accept the suggestions or select another domain).

*CBT* cognitive–behavioral therapy, *PF* psychological flexibility, *ACT* acceptance and commitment therapy
